# “Patients’ interests first, but … ”–Austrian Veterinarians’ Attitudes to Moral Challenges in Modern Small Animal Practice

**DOI:** 10.3390/ani9050241

**Published:** 2019-05-15

**Authors:** Svenja Springer, Peter Sandøe, Thomas Bøker Lund, Herwig Grimm

**Affiliations:** 1 Unit of Ethics and Human-Animal-Studies, Messerli Research Institute, University of Veterinary Medicine, Vienna, Medical University of Vienna, University of Vienna, 1210 Vienna, Austria; Herwig.Grimm@vetmeduni.ac.at; 2 Department of Food and Resource Economics, University of Copenhagen, 1958 Frederiksberg C, Denmark; pes@sund.ku.dk (P.S.); tblu@ifro.ku.dk (T.B.L.); 3 Department of Veterinary and Animal Science, University of Copenhagen, 1870 Frederiksberg C, Denmark

**Keywords:** advanced veterinary medicine, small animals, focus group study, veterinary medical ethics

## Abstract

**Simple Summary:**

Hip arthroplasty, heart valve replacement, dialysis, and specialties such as oncology, cardiology and neurology are becoming standard in modern small animal practice, which, in some respects, is not far behind the field of human medicine. This focus group study of veterinarians (*n* = 32) examined the effect of these advances and the challenges they introduce. The study shows that while modern diagnostics and therapies deliver benefits in patient care, they also add complexities to decision-making. Although the veterinarians participating in the study were aware of their duty to act in the best interests of the animal, their decisions were highly dependent on factors such as the client’s financial background and the emotional bond between client and animal, as well as the veterinarian’s place of work, and level and field of specialization, and certain economic aspects of the practice. The overall conclusion is that veterinarians are increasingly torn between patients’ interests, medical feasibility and factors related to the client, the veterinarian, and professional colleagues. The findings also suggest that services are not only oriented towards the provision of medical care in a strict medical sense. On top of this, veterinarians need to deal with various expectations and wishes of clients which influence their decision-making. As it will be shown, factors like the possibility of referring patients to specialist veterinarians or prompt diagnostic results influence their decision-making.

**Abstract:**

Small veterinary practice is experiencing steady improvement in diagnostics and therapies which enable veterinarians to offer evermore advanced medical care for their patients. This focus group study of veterinarians (*n* = 32) examined the impact of these improvements and the potential challenges they introduce in small animal practice. It shows that while advanced diagnostics and therapies deliver benefits in patient care, they also add complexities to decision-making. Although the veterinarians participating in the study were aware of their duty to act in the best interests of the animal, their decisions were highly dependent on factors such as the client’s financial background and the emotional bond between client and animal, as well as the veterinarian’s place of work, and level and field of specialization, and certain economic aspects of the practice. The overall conclusion is that small animal veterinarians are increasingly torn between serving the best interests of the animal, medical feasibility and contextual factors related to the client, the veterinarian, and professional colleagues. Further, the findings suggest that services are not only oriented towards the provision of medical care in a strict medical sense. On top of this, veterinarians need to deal with various expectations and wishes of clients which influence their decision-making. As it will be shown, factors like the possibility of referring patients to specialist veterinarians or prompt diagnostic results influence their decision-making.

## 1. Introduction

In recent years, there have been significant diagnostic and therapeutic advances in small animal medicine. Veterinary oncologists can now diagnose cancer in companion animals with advanced imaging techniques, like computer tomography [1], and then treat it using therapeutic methods, such as chemotherapy and radiotherapy. Hip arthroplasty, heart valve replacement, dialysis, imaging, and specialties, such as cardiology and neurology, are becoming standard. In short, modern companion animal veterinary medicine provides many treatment options that are not far behind those offered in human medicine. For example, cancer in small animals can not only be detected at an earlier stage with advanced diagnostic imaging techniques like computer tomography [1]; it can also be treated with various therapeutic methods, such as chemotherapy and radiotherapy, which are increasingly being used to prolong the lives of affected animals.

There is no doubt that animal patients can benefit from improved diagnostics and therapies if they are used in the animal’s best interests. To act in the best interests of an animal can be understood as a matter of promoting its health and improving its quality of life [2]. In the literature proper consideration of these patient-centered factors is often presented as fundamental [3,4,5], and some authors make a normative claim in favor of a strong patient advocacy in companion animal medicine [6,7,8,9,10]. For example, the philosopher Simon Coghlan points out that the significant status of companion animals and the role-based duties of veterinarians to their patients combine to justify the prioritization of the best interest principle during patient care. He concludes that veterinarians “have ‘primary obligation’ and ‘first allegiance’ to their animal patients rather than to other parties, such as their clients or employers” [9].

However, recent empirical investigations question whether this view is realistic and indicate that decision-making processes in small animal practice are strongly influenced by contextual factors related to the client, the veterinarian, professional colleagues and the working environment [11,12,13,14,15,16]. This can create moral dilemmas for veterinary professionals [17]. The contextual factors may collide with the veterinarians’ supposed advocatory role, and when they do it will be morally challenging for the veterinarian to comply with the best interest principle.

This can be seen in a study conducted in the UK, where small animal veterinarians indicated that financial limitations, the clients’ preference to continue treatment, and convenience euthanasia were creating ethical dilemmas in veterinarians’ daily working life [14]. Other empirical studies have highlighted the importance of upfront discussions of costs during patient care [15] and looked at the complexities of dealing with financially limited clients [12]. The handling of such clients is perceived as especially stressful [11,14], as this restricts the provision of best care to the animal patient, but also has negative effects on the veterinary clinic’s own financial viability. The combination of economic factors and veterinarian’s position as a service provider is also important, and is often-discussed. For example, in 2012 the Austrian Institute for Economic Research published a report on the economic basis for strategic decisions on future aspects of veterinary medicine. The report shows that veterinary medicine is predominantly a service provider, and that it, therefore, depends closely on the development of downstream sectors and private demand [18]. In light of this, veterinarians are obliged to consider economic aspects and act as service providers at the same time. 

In cases where clients have a strong emotional bond with their animals, the client’s expectations and wishes can lead to especially complex and emotionally driven decision-making. A study of companion animal ownership conducted in Austria found that strong emotional bonds between Austrians and their pets had become more common in the period 2012–2017. In 2017, of a total of 1009 interviewees (90%) stated that they see their dogs and cats as good friends (up 7% since 2012). In all 74% of respondents revealed that their animal has the status of a family member (up 9% since 2012) [19]. 

Strong relationships become more complex if the animal contracts a disease and its care becomes a task for the owner [20]. In a US study, veterinarians agreed that giving due consideration to the bonding between clients and their animals has a positive effect, and that they are more successful when they recognize and facilitate the human-animal-bond [16]. By contrast, some authors associate strong human-animal bonds with negative consequences such as overtreatment and prolongation of animal suffering as a side-effect of the owner’s request for further therapy [21,22,23,24]. 

These findings make it clear that it can be a challenge for veterinarians to maintain their role as an animal advocate in veterinary practice. With more advanced, and often more costly, treatments, the challenges may intensify. Arguably, this development has been coming for many years: already, in 1995, Tannenbaum stated that the intensity and severity of ethical challenges would increase with the establishment of advanced methods and technologies in veterinary practice [25]. Additionally, he argued that the issue of overtreatment and client finance and time limits would confront veterinary professionals with especially difficult ethical issues. 

It should also be borne in mind that the veterinary profession appears to be witnessing growing specialization—a development which can be partly ascribed to the ever-growing implementation of advanced technologies and methods [26,27]. Although specialization brings benefits such as improved knowledge, it also potentially generates issues between involved parties [27]. The existence of different knowledge bases, and specialists with their own expertise, leads to an increasing compartmentalization of the profession, which, in turn, can introduce different, or even conflicting, interests in the course of veterinary treatments. Disagreements can emerge between specialist veterinarians working in different fields at the same clinic, and of course they can also arise between general practitioners and specialist veterinarians in the course of the referral process.

The hypothesis of the present paper is that advanced diagnostics and therapies in small veterinary practice add to the complexity of decision-making during patient care. To the authors’ knowledge, the assumption that the number and severity of ethically challenging situations in the veterinary practice increases as a result of use of advanced diagnostics and therapies has not yet been verified empirically in a systematic way. It is the purpose of this paper to fill this gap in our understanding of veterinary practice. 

A focus group study with small animal veterinarians was carried out in Austria to gather insights into this issue. Veterinarians working in Austria offer a suitable study population for this explorative study, since Austria offers a variety of business models within the veterinary profession. Thus, besides the university hospital and small practices, Austria has both corporate chains of clinics, like those in the UK, and privately-owned clinics of various sizes.

A qualitative method was chosen because no studies to date have looked at advanced veterinary medicine and the challenges it introduces in practice. Focus group discussions, unlike other qualitative methods such as semi-structured individual interviews, also allow the differing attitudes and beliefs of veterinarians to be revealed more fully if there is a lively debate on everyday challenges in the profession. In addition, the mutual exchange of views helps to pinpoint not only commonalities, but also differences in opinion and uncertainties among participants [28,29]. 

The aims of the study were: first, to identify the patient-centered factors which veterinarians see as relevant during patient care; second, to investigate other contextual factors that influence decision-making processes during patient care in general; and third, to explore these other factors and their effects in the specific circumstances of advanced veterinary practice. 

## 2. Materials and Methods 

A total of six focus group discussions with 4–7 participants, all of whom were small animal veterinarians, were conducted in Austria in March and April, 2018 (*n* = 32). 

### 2.1. Overview of the Number of Austrian Small Animal Veterinarians and the Selection of Participants

Approximate numbers of veterinarians, practices, and clinics in Austria working with small animals were determined using multiple sources, whereas practices differ from clinics by the number of veterinarians (practices: 1–3 veterinarians; clinics: more than three veterinarians) and specialization (practices: generalists and basic equipment; clinics: higher degree of specialization and more advanced equipment). Thus the Austrian Association of Small Animal Veterinarians (VÖK) (register contains: 1327 veterinarians/status Nov 2017), a large pharmacy group (list of 792 small animal practices and clinics working with small animals/status Nov 2017) and a comprehensive search of an online classified directory (778 Austrian practices and clinics working with small animals were found/status Nov 2017) provided an overview of veterinarians, as well as practices and clinics in the field of small animal medicine.

The six focus group discussions were planned so as to include veterinarians with various positions and roles in practices and clinics in the small animal sector in Austria, as we hypothesized that these different positions may affect attitudes and decision-making. Thus, the sampling strategy involved participants from several strata: a) current occupation—divided according to hierarchical position (practice owner or employee) or level of specialization; b) availability of equipment; c) number of colleagues; d) degree of urbanization (from rural region up to capital city (Vienna)); and e) working place (federal state). Recruitment to the focus groups was organized so that the groups were homogenous with respect to the current occupation of the participants (Table 1). Group 1 and Group 3 included specialist veterinarians working at the university hospital in Vienna and at several referral clinics in Austria, respectively. Group 2 included managers and clinic owners. Groups 4, 5, and 6 contained general practitioners who were self-employed with no or very few employed veterinarians. Further details regarding the distribution of the four other factors (i.e., those listed in b), c), d), and e) above) within and between the groups are set out in Table 1. Veterinarians were only recruited due to their involvement in small animal medicine. Thus, out of the 32 veterinarians, 30 participants worked solely with small animals. Only one veterinarian also worked with farm animals and one participant also worked as an official veterinarian. 

### 2.2. Recruitment Process and the Study Participants

In mid-January 2018, invitations were emailed to 60 selected veterinarians on the basis of the above-mentioned criteria with the aim of including 10 veterinarians in each group. All of the veterinarians were personally contacted by telephone two weeks later, at the end of January 2018. Suitable replacement candidates were selected if veterinarians declined the invitation or did not react within two weeks of the emails being sent out. The invitation and recruiting process was closed when at least five participants per group had confirmed their participation: this turned out to be at the end of February for Groups 1–5 and end of March for Group 6. Reminder emails were sent to all participants 3–4 days before the scheduled focus group discussions. In total, 114 veterinarians were contacted during the recruitment process, and 36 confirmed their participation. Four veterinarians could not participate owing to unforeseen events (three were ill and the car of the fourth broke down), so ultimately 32 veterinarians participated. Table 1 provides a general overview of the participants in all six focus groups. The veterinarians did not receive an allowance or an honorarium. However, they were informed that their travel expenses would be reimbursed.

### 2.3. Structure of Focus Group Discussions

All six focus group discussions were conducted in German. The shortest was 2 hours and 24 minutes. The longest was 2 hours and 37 minutes. All discussion in the groups followed a semi-structured interview guide. This consisted of four themes structured according to the so-called ‘funnel approach’, i.e., it started with general questions about patient care in veterinary medicine and moved on to more specific questions. Depending on the group, the formulation of individual questions was slightly changed and adapted to suit the participants and their working background (Supplementary Files 1–3). However, the process of each group discussion was the same in terms of structure and overall content. Throughout the discussions, the moderator, who ensured that all participants had the opportunity to speak and be heard, took a neutral position.

The steps and the contents of the interview guide were piloted on four veterinarian test persons. These test runs did not aim to simulate a group discussion. They were designed to evaluate and verify the clarity of the interview guide questions and topics, and to see whether the questions triggered relevant thoughts and responses. Comments and suggestions made by the test persons were incorporated into the final versions of interview guides. 

After a short introductory round (min. duration: nine minutes; max. duration: 28 minutes), the first part of the interview guide aimed to elicit an overall impression of important aspects of patient care in small animal practice (Theme 1). Further, participants were required to order the interests of each of the three key stakeholders: veterinarian, animal, and client. Challenges related to the clients, and their expectations given the diagnostic and therapeutic possibilities, were identified (min.: 26 minutes; max.: 47 minutes). The second part of the interview guide aimed to direct the discussion towards the topic of advanced diagnostic and therapy in veterinary medicine by identifying related uncertainties (Theme 2). Additionally, participants were asked to describe cases where they thought veterinarians go too far in diagnostics and therapy (min.: 29 minutes; max.: 48 minutes). During the third part, veterinarians were introduced to three newspaper headlines which brought out the issue of advanced diagnostics and therapy in veterinary medicine in a thought-provoking way (Theme 3). Using the newspaper headlines, the discussion was elevated from the veterinarian-client-animal context to a social context and social discourse. The overall aim was to identify attitudes to the headlines, and to explicate possible divergent attitudes to advanced methods in relation to specific fields of specialization (min.: 15 minutes; max.: 28 minutes). The fourth part was structured around three designed case vignettes. These aimed to elicit responses to different moral dilemmas occurring in the context of advanced veterinary medicine (Theme 4). The aim was to see how veterinarians would handle such situations, and the extent to which factors relating to the animal, the client’s characteristics, and the veterinarian, as well as the working environment, determined decision-making processes (min.: 29 minutes; max.: 37 minutes). 

In addition to the moderator (HG), a second person from the project (SS) was present at each discussion to take notes and, if necessary, ask interposed questions. Before recording started, each participant signed a consent form and was informed that all recordings would be treated confidentially. Ethical approval was obtained through the Ethics Committee of the Medical University of Vienna.

### 2.4. Data Analysis

Recordings of all six focus groups were transcribed verbatim and coded using the MAXQDA 12.0 software program (Berlin, Germany). Following the template organizing style [30], categories and codes were formulated based on the four themes and key aspects of the interview guide, the research questions and hypotheses of the project. A first cycle of coding and a second cycle of coding were conducted for the analysis of the data gathered from the six focus groups [31]. Using a deductive approach, the overall aim of the first cycle of coding was to summarize segments of data and categorize similar data units [31]. The initial code list for the first cycle contained six categories with a total of 24 codes. During first cycle of coding this initial list was adjusted: new codes and categories were added, and other codes that had become non-applicable or redundant were deleted. These alterations chiefly followed an inductive approach based on the gathered data. The final version of the code list included seven categories with 28 codes (Supplementary File 4). Mainly descriptive coding, hypothesis coding and holistic coding were used to organize and summarize the text segments [31]. The coding process was mainly done with the transcribed text. Only if uncertainties arose (e.g., concerning negative or positive meanings of statements), the coder (SS) resorted to the recordings. Codes and ascribed segments of data were continuously discussed by the project team (SS, PS, TL, and HG) in order to ensure the relevance of codes, especially during the first coding cycle. In a second cycle of coding, the initial results of the analysis were grouped into smaller categories and clusters to obtain more meaningful units for subsequent content analysis [31]. This was a qualitative social science study where it is not an ambition to make statistical generalizations about a background population. Rather, the aim is to obtain open-ended and in-depth accounts of beliefs, attitudes, everyday practices, and decisions.

## 3. Results

The focus-group study identified two main groups of factors affecting veterinary care of small animals. First, there were patient-centered factors. These were widely seen as relevant to patient care in small animal practice. Second, there were other contextual factors. These appear to influence decision-making processes in veterinary practice in general. They also seem to have grown in importance as a result of the development and implementation of advanced technologies and methods in modern small animal practice. 

### 3.1. Patient-Centered Factors

The aim at the beginning of all focus group discussions was to identify the most important aspects of patient care. Several participants mentioned factors connected with their own competencies and qualities, as well as the importance of acting in the animal’s interests. On the basis of patient-centered factors, veterinarians highlighted their role as advocates for the animal patient.

#### 3.1.1. Medical Competencies and Professional Quality

All participants in the study indicated that their veterinary competence and the quality of their professional work are basic prerequisites of patient care. They talked about the general importance of treating the animal to the “*best of their knowledge and belief*” and providing “*state of the art*” therapy. For instance, one veterinarian stated that “*when a patient is in my care it is important to me that I, as a [small animal] doctor, feel I have the competence and knowledge to provide the best possible care.*”

#### 3.1.2. Acting in the Best Interest of the Animal

The veterinarians also referred to their awareness of their role as advocates for their patients. One stated that a veterinarian has to *“speak for the patient, because [the animal] herself cannot speak up.”* They consistently expressed concern about the animal’s quality of life and their commitment to promoting the patient’s well-being. A group of veterinarians with extensive professional experience said that the idea that the patient should come first is part of the self-understanding of the profession. They suggested that this priority is the reason why veterinarians study veterinary medicine.

These statements were echoed by a specialist veterinarian, working in a referral clinic, who emphasized the importance of focusing on the animal during patient care, and who stated that the animal patient, not its owner, should be considered the “client”. However, the veterinarian toned down this statement, adding: *“Oftentimes, I have to compromise a lot more than I would want to, because [the client] has the power to decide. But if you ask me who I want to please: the patient, not the client.”* Discussions in all of the groups moved on rapidly to the impact of other contextual factors on decisions. 

### 3.2. Other Contextual Factors and Effects

Although veterinarians are aware of their advocatory role and mentioned that patients’ interests should be prioritized, it became obvious that they encounter other demands which limit their ability to focus solely on the best interests of the animals. At the start of each focus group, general questions about patient care enabled the interviewers to identify contextual factors which seem to be of importance in veterinary practice in general. Client-centered factors were especially visible during this part of the discussion. 

#### 3.2.1. Clients’ Ability to Understand 

The significance of differences between clients was touched on in all of the focus group discussions. Participants often reported that they needed to deal with clients flexibly—for instance, by speaking in a language understandable to them. A veterinarian working at the university hospital said: *“you open and close the door and find yourself in a completely different situation. [...] While I was just able [previously] to use Latin terms, the next moment I have to try to somehow explain to someone what the complex disease of their animal means as simply as possible […].”*

#### 3.2.2. Clients’ Ability to Pay

The need to respond flexibly to the financial status of the client was also raised in every focus group discussion. Participants indicated that financial limitations often forced them to find the best possible diagnostics and treatment options within a budget. Veterinarians working in urban areas stressed this point especially strongly. One, working as a general practitioner in Vienna, stated: *“I have to actively pay attention to the clients, and find out whether they can afford my service […]. Clients are at their limits very often. They are at their limit and there are only a few clients for whom money doesn’t play a role, so to speak.”* Another veterinarian working in Vienna related: *“Well, for me—I don’t come from any noble district—money is a very, very big issue. I have a lot of pensioners on minimum rate, and that’s where I have to make up my mind. […] I mean, there are many who don’t want to afford it, but many also can’t afford it.”*

#### 3.2.3. Emotional Bond between Client and Animal

Again, in all focus groups participants mentioned the issue of over-treatment resulting from the tight emotional bond between the client and their animal. Conflicts between the clients’ wish to continue treatment at any cost versus the veterinarians’ responsibility to discontinue treatment and end the animal’s suffering were a common problem in veterinary practice and perceived as rather challenging. One veterinarian explicitly stated that in her view a problematic “human-animal-bond” leads to these challenging situations and pointed out that *“it is actually misplaced love for their animals when people say they want to extend it. [...] I mean it is misguided love towards the animal to just think, I want to do everything, when actually there’s no way out.”*

#### 3.2.4. Client Satisfaction and Respectful Relationships

Veterinarians in all of the focus groups frequently referred to the importance of a respectful relationship with the client, and securing client satisfaction. It was noted especially that trust, and the ability to empathize with clients, led not only to client satisfaction, but also to positive recognition of veterinarians’ work.

Client satisfaction was a topic that clinic owners focused on particularly. One owner stated: “*if the client is satisfied, it’s good for the animal. [...] Then it’s okay for me and the sale of the service fits. And the employees are also satisfied, because a satisfied client certainly radiates well-being […].*” A further aspect of the significance of good veterinarian-client relationships was the idea that it is easier (or perhaps only possible) to act in the best interests of the animal by acting together with the client. For example, one veterinarian indicated that “*[the animal and the client] are a team, yes, and basically I can reach the welfare of the animal only through the clients […] because they are the ones who make and pay for the decisions and who instruct me.*”

### 3.3. Contextual Factors and Advanced Small Animal Practice

It was a key aim of the interviews to investigate how recent advances in small veterinary medicine have influenced decision-making in patient care. Several implications of modern small animal practice received attention in the focus group discussions, with connections being made with the animal, the veterinarian, the client and the veterinary profession in general.

#### 3.3.1. Improvement in Patient Care and the Patient’s Quality of Life

Across all focus groups, veterinarians indicated that new technologies and methods were providing better insights into the health status of animal patients and improving therapies. A specialist veterinarian stated: *“Yes, I actually see development in the sense of improvement. […] I actually see further development as desirable and a standstill is actually out of the question.”*

Although technologies offer improvements in patient care, several veterinarians insisted that the quality of the patient’s life comes first and should be prioritized over the prolongation of life. An oncologist referred to his advocatory position and declared: *“[…] we can still extend [life] by a few weeks, but for a price that is not justified. I’m talking about quality of life for the animal. That’s certainly the daily challenge in the field of oncology, to say: Stop! Until here and no further. Saying, as the voice of the patient: Not everything that can be done makes sense.”* Veterinarians working in small practices echoed this statement. A general practitioner stated: *“it is also good that we have these possibilities, but the most important thing is the quality of life, […] that we foreground the animal, and we don’t need to exploit every diagnostic possibility with high-tech at any price.”*

#### 3.3.2. Technology as a Motivating Factor 

Veterinarians indicated that new technologies and other developments in the profession motivate them in their daily working life. Thus one veterinarian, working at the university hospital, said: *“I see medical progress as [...] a very important motivation for further training, for everyday working life, where one can basically get a sense of achievement from patient care. I think that is a very important point—if you always stayed at the same level, it would be boring somehow.”* This sentiment was not only expressed by the specialist veterinarians. The study participants working in small practices were also energized by self-improvement: *“I think it’s very important that we constantly educate ourselves and apply new methods that have already been tried and tested by clinics and universities […]. And I also think it’s important for us to get input from time to time, because then the work is more fun again. Just learning something new.”* In addition, some veterinarians indicated that the presence of certain technical devices leads to advantageous simplification of their work—saving them time or giving them the opportunity to work independently of other practices or service providers, e.g., laboratories. 

#### 3.3.3. Disproportionate Use of Diagnostics and Therapies

Many of the veterinarians were worried about the disproportionate use of diagnostics and therapies. It became apparent that they see different reasons for this problem, including financial drivers on the veterinarian’s side, inexperience and the “hedging” by younger veterinarians, veterinarians’ “blindness” when focusing on isolated aspects of the patient due to specialization, and client preferences for further diagnostics and therapies for their animal.

At this point, differences between general practitioners and specialists emerged. All three focus groups with veterinarians working in small practices agreed that some clinics—particularly the larger ones—conduct unnecessary double diagnostics or diagnostics for cases with a poor prognosis. By contrast, however, a specialist veterinarian working at the university hospital stated that *“if I have a client who says they want the best possible medical care, then it is not so easy to balance between fancy technique and simple intervention. […] And if I want to have the greatest possible certainty—want to have it as a client—well, then I also need full diagnostics.”* Further, clinic owners participating in the study mentioned that additional diagnostic investigations improve their legal security because they provide documentation. They stated that the documentation offers a form of hedging that can be valuable in cases where possible post-operative complications can occur.

#### 3.3.4. Growing Client Expectations and the Internet

The focus group participants indicated that clients’ expectations had risen as a result of new technologies. However, different factors emerged as salient here, depending especially on the working position occupied by the veterinarian and their background. The clinic owners strongly connected the client’s expectations with the sophistication of the technologies being used. For example, one owner indicated that *“it also impresses the client when he sees several things in color, sees them three-dimensionally. [...] The processing—that you film it, archive it, show it to the client. That’s quite important, to sell our service, right?”* Another stated that clients now expect diagnostics and results to be delivered immediately: *“people not only expect us to do everything and that we are able to do everything, but also they want it to happen immediately and today and right away, right? [...] And that’s why you’re forced to push these technical things up to this level!”* In contrast, a veterinarian working at the university hospital cited developments in human medicine and the patient’s status as a family member as reasons for rising client expectations about patient care in small animal practice. The veterinarians working in small practices indicated that their clients were increasingly making use of specialists. 

The participants discussed the fact that clients choose veterinary practices by looking at the range of technical devices being offered, and the fact that information about practices and their services is increasingly exchanged by clients via the internet and social media channels. Clients who inform themselves about issues on the internet in advance, and who try to treat their animals themselves, were regarded as especially challenging. One veterinarian reported: *“Dr. Google was asked. And now, [the client] wants this and that. They have already treated [the animal] with home remedies for three weeks and it has not helped. Now, [the client] wants to have an injection for [the animal], and then everything is good.”* Another pointed out that *“nowadays, there is Dr. Google. Hence, [the clients] question everything or they already come with diagnoses from the internet. And they are very unpleasant if you don’t confirm this diagnosis.”*

#### 3.3.5. Advantages of Referring

Participants in all of the focus groups referred to the advantages of referring patients. The general practitioners and specialists obviously had somewhat different perspectives on these advantages. Veterinarians working in small practices know their limits and feel relief that they can refer their patients to specialist veterinarians. One said: *“when it comes to new techniques and the like, I feel like a general practitioner and I cannot be up to date with the latest technique, because it is very specialized. I can’t have the knowledge of the university […].”* Thus, referring patients reduces the uncertainties of veterinarians working in small practices. However, several general practitioners working in small practices also mentioned that exchanges with specialist colleagues, together with a trustful relationship with them, are crucial and facilitate the process of referral.

#### 3.3.6. Specialization and Institutional Background 

There was a general agreement among all groups that the new diagnostic and therapeutic technologies require time and specialist knowledge. Specialists mentioned that their knowledge and expertise made them more secure in their use of the new technologies and methods. Veterinarians working at the university frequently emphasized that their special institutional background—their employment at the university—provides better financial security than that offered by privately-owned practices. The veterinarians working at the university hospital observed that it takes a special personality to work at the university and to get used to dealing with the new technologies: *“I think if you’re at that level, we can handle new equipment like that, right? And I think that’s our job as the university, right? So, we are not a practicing veterinarian, who perhaps gets it ten years later […].”*

Several of the participants (general practitioners and specialists) pointed out that the institutional background combined with specialized knowledge justifies the use of technologies during patient care. For example, one specialist said: *“The question is whether I’m in an environment that provides justification for using these new techniques, because I have them at my disposal—on the one hand. On the other hand, of course I may already know what I can use these techniques for.”* A general practitioner agreed about this, stating that *“a clinic does more diagnostics with a certain right, which I cannot do in my small practice. Firstly, because I don’t have the means. Secondly, because it is not possible with my cost structure and the way my clients see me […].”* It can be seen, then, that there was felt to be a distinction between general practitioners and specialists—one based mainly on differences in specialist knowledge and institutional background which were, in turn, connected with, and required, certain kinds of personality.

#### 3.3.7. Financial Pressure

It became apparent that new technologies can create financial challenges. One veterinarian, for instance, remarked that “*the financial component plays a decisive role when it comes to this modern device. Basically, it is simply a problem that we have to offer multi-tier health care systems. We have treatments that one person can afford, but another cannot.*” Another colleague at the university said: “*unfortunately, sometimes modern therapy methods don’t come cheap. That’s why it’s a great pity if you always have to combine the medical aspect with economic aspects in some way. As a doctor you actually want to talk more about medicine and less about money.*” Only a few veterinarians mentioned insurance for animal patients as a possible solution, and when they did, they indicated that insurance plays a very small role in Austria as compared with its use in other countries.

#### 3.3.8. Amortization of Technical Equipment, Profit, and Market Competition

Several focus group participants in addition to referring to client finances, also mentioned the financial goals and requirements of veterinarians and their clinics. One recurring factor was the problem of amortization of technical devices—a process which can lead to over-use of diagnostics and therapies in order to make profit.

As one veterinarian said: “*basically, the expensive diagnostics—there I see the problem that, of course, it has to be amortized and financed. I also see the problem of exaggerated diagnostics and, how can I say this, too frequent use, where it may not be necessary, because it has to be amortized.*” By contrast, one veterinarian working at the university hospital saw herself in a privileged situation: “*I think we have the huge advantage that we have a very large institution behind us, namely the university. […] And we don’t have this immediate pressure to use a new device immediately on the patient right away.*” 

Another challenge—one which attracted attention during focus group discussions and appeared to be closely related to owner expectations—was set by the fact that realistically clinics have to be competitive. Clinic owners were especially likely to mention that they felt under pressure to buy new advanced equipment in order to provide care of the highest standard. One indicated that *“the pressure doesn’t come from the device hitting the market, but from the clinic’s need to remain competitive. Of course, if other clinics around offer something else that you can’t do at all, you have to do something.”*

## 4. Discussion

Our current understanding of the various effects of implementation of new methods and technologies in veterinary medicine is largely anecdotal. This is the first time that empirical findings on the veterinary experience have been collected and analyzed in a way that helps us to understand: a) the patient-centered factors which impact on veterinarians during patient care; b) the other contextual factors influencing veterinary decision-makings in general; and c) specific knock-on effects of advances in small animal practice. In this section, the findings of the present study are considered in relation to relevant published literature. It is pointed out where our data support or add to existing knowledge and where they contrast with earlier findings or assumptions.

The normative claim that veterinarians should prioritize the animal patient’s interests in decision-making processes has been a central concern in recent literature [5,7,10]. Empirical findings also confirm that veterinary clients support the notion that it is the role of the veterinarian to advocate in the patient’s best interests. Thus, Hughes and colleagues found in their study of British and Australian clients that animal owners consider a commitment to the patient’s welfare and quality of life to be the most important goal that veterinarians *should* pursue [32]. However, a question arises about the extent to which professionals *can* adhere to such normative demands under everyday clinical circumstances [33,34]. Although our results indicated that modern developments in veterinary medicine offer increasingly effective ways to serve the health interests of animals with improved levels of patient care, we also found that a number of contextual factors and effects of advanced medicine push in the opposite direction and prevent veterinarians from focusing solely on the best interest of their patients, and that these factors generally lie beyond the veterinarian’s own control. 

The participants of all of our focus groups confirmed that clients with limited funds present an everyday challenge in the veterinarian’s working life. This is in line with other studies, which show that the issue of the client’s ability to pay is a frequent problem and creates economic, as well as moral, challenges for professionals [11,12,14,15,35]. For instance, in a focus group study conducted in the US, Coe and colleagues considered financial aspects of animal patient care with invited veterinarians and clients [15]. Their results show that veterinarians find it challenging when patient care is highly dependent on the financial situation of the client and, connectedly, his or her willingness to agree on treatment. Our findings add to this conclusion. They indicate that the implementation of cost-intensive methods and technologies exacerbates the problem presented by clients with limited funds in modern small animal practice, and that veterinarians are worried about their increasing dependency on clients with sufficient money to pay for sophisticated procedures.

Interestingly, Kogan and colleagues found that most of the veterinarians they surveyed offered discounts on veterinary services and products on a regular basis in order to provide the best possible solution and care for their patients [36]. This finding was not confirmed by our study. Although the participants in the focus groups we ran explained that they work increasingly in a multi-tier health care system due to the increase of cost-intensive diagnostics and therapies, they focused mainly on finding solutions adapted to the financial situations of the client—they did not, in other words, provide discounts or pro-bono services on a regular basis. This may be connected with the veterinarians’ own financial needs: it may be that they are simply seeking to fund the purchase of expensive technical devices through client payments. A further reason could be that veterinarians see a risk that clients will exploit their good nature and keep asking for reduced prices—the news will likely get around and encourage those requests.

It was expected that in the focus group discussions, the issue of how to finance advanced diagnostics and treatments in small animal practice would be closely tied to questions about pet insurance. However, this was not confirmed: the topic of pet insurance was not at all prominent in the discussions. This may be due to the fact that, in Austria, the market in pet insurance is relatively undeveloped. It is estimated that the number of insured animals is (far) below 10% [37]. This is in stark contrast with the situation in other countries. For instance, almost 80% of dogs in Sweden are insured [38]. In the UK, in 2017, there are about 80 providers of pet insurance and demand is increasing [39].

The market for pet insurance in Austria is surely set to grow. Since little research has been conducted to date on the impact of insurance on veterinarians’ daily working life, further investigation into the extent to which Austrian pet insurance might deal with the problem of clients with limited funds, leading to relief not only on the client’s side, but also for the veterinarian, is certainly needed. Interestingly, Coe and colleagues found that US veterinarians were worried that American pet insurance has different programs covering different kinds of veterinary cost—the speculation was that this could indirectly influence veterinarians’ provision of patient care [15]. This potentially adverse effects of pet insurance needs to be considered. Cooperation between insurance providers and veterinary associations might offer a suitable way to deal with the concerns mentioned by US veterinarians during the process of market introduction. 

Turning to the veterinarians’ discussion, in our study, of clients’ levels of knowledge, use of the internet and related expectations, our data suggest that the internet and social media are indeed having an impact on veterinary practice. The ready availability of information via the Internet leads to high information flow and causes different expectations on the clients’ side [40]. In line with Clarke, our results indicate that client research using “Dr. Google” puts pressure on veterinarians and challenges them during consultations [41]. Relatedly, Knights and Clarke have found that the availability of various media sources means that veterinarians are subjected to a high degree of judgment and assessment—their performance is constantly evaluated by “others” [42]. The veterinarians surveyed by Knights and Clarke expressed concerns especially about complaints via “virtual channels”. Given these empirical findings, we suggest that social media, websites, and the use of “Dr. Google” should play a larger role in future debates about modern veterinary practice. There is an increasing need to use the internet and other new media to meet client expectations—to market veterinary services and alert clients to special offers, for example. At the same time, veterinarians must consider how best to deal with inflated or skewed expectations among clients caused by the combination of new technical possibilities and the unfiltered information flow from the internet. 

In keeping with previous studies [14,16], the knock-on effects of close emotional bonds between clients and their animals were frequently discussed in all of our focus groups. Although such bonds can have a positive effect during patient care—for example, because clients are more willing to pay for costly diagnostics and therapies [43]—we were not surprised to find that they can also lead to problematic overtreatment. Our analysis shows that the problem of overtreatment is accentuated by new technologies and methods, and is perceived as quite challenging. At this juncture, veterinarians working at the university hospital explicitly mentioned that the status of patients as family members, as well as close emotional relationships, leads to higher client expectations about the care of their animals. Presumably, veterinarians working in well-equipped clinics are particularly likely to be confronted with such increasing client expectations, because they can deliver the highest levels of treatment. 

A further insight given by our findings relates to the process of referring patients and the increasing specialization of the veterinary profession. Relatively few studies [44,45,46,47] have examined various factors in the veterinary referral process. A recently published focus group study with equine veterinarians found that the relationship between the client and the referring veterinarian, the involvement of the referring veterinarian, good mechanisms of communication, and a collegial relationship between the general practitioners and specialist, are crucial aspects for a good referral process [44]. The small animal veterinarians also indicated that a good and trustful relationship is fundamental to a good referral process. However, our results go further when it comes to advances in veterinary medicine. For they reveal that both general practitioners and specialists believe that that the use of modern technologies and methods increasingly requires detailed knowledge and time. Those working as general practitioners indicated that they felt relief when referring patients to specialists in cases where the limits of their equipment and professional knowledge were being tested. Such a back-up from a university or a referral clinic leads to more security in general practice by allowing practitioners to define clear limits to their competencies. Hence, veterinarians were aware of their different roles and responsibilities, and the relations between these and their own degrees of specialization and institutional backgrounds. 

A key hypothesis of this study was that advanced veterinary medicine adds to the complexity of decision-making, and that this complexity explains in part why patient care is experienced as morally challenging by veterinarians. We found that veterinarians are particularly likely to face moral challenges where they are not able to comply with patient-centered demands. We do not, of course, dispute the normative claim that small animal practice should be patient-centered, and that veterinarians should play an advocatory role. Our point is rather that the findings presented in our study suggest that veterinarians cannot always follow this norm in their daily work. We see a danger that a moralizing approach to small animal practitioners claiming that they *must* give priority to the best interest principle during animal patient care will simply increase levels of stress within the profession without really helping any patients. 

Although it is a strength of the present study that participants in it were carefully selected through a thorough search of the Austrian population of small animal veterinarians, the study also has limitations. The focus group method can create bias when strong opinion-makers in the group dominate the discussion. Additionally, the moderator may steer the discussion, knowingly or unknowingly, in a direction which delivers desired responses, or avoids unwanted outcomes. In the present study, we attempted to minimize bias by conducting several discussion groups, by instructing the moderator to follow an interview-guide, and by ensuring that the moderator was accompanied by a second person from the project, who observed procedure and thematic development. The fact that the study was carried out in Austria may also mean that some of the results will be less relevant in other countries, because of cross-country differences in the institutional set-up of small animal practices—the comparatively low rate of pet insurance in Austria is an obvious example here. 

Focus group studies are explorative in nature: they offer an opportunity to generate hypotheses rather than test them. Therefore, we recommend that a quantitative study—e.g., a questionnaire-based survey—should be undertaken to provide representative results relating to the themes presented here. Additionally, further research on this subject could focus on cross-country disparities. It could compare the effect of different legal backgrounds, different organizational structures in small animal practice (e.g., private practices and corporate chains), and different professional norms, as these emerge in national comparisons.

## 5. Conclusions

The results of the present focus group study support our speculation that although advanced diagnostics and therapies lead to real benefits in patient care in modern small animal practice, they also add complexities to the decision-making process, bringing in new contextual factors and creating moral challenges. The challenges arise particularly from veterinarians’ dual awareness (a) that they are advocates for their patients, and (b) that their decisions are highly dependent on client-centered factors, such as the client’s financial background and the emotional bond between client and animal. Further, there is evidence that service provision is not only oriented towards the provision of medical care in the strict technical sense: veterinarians need to deal with the expectations and wishes of clients, and increasingly these client-centered factors influence veterinary decision-making.

## Figures and Tables

**Table 1:** Details about focus group participants and composition (*n* = 32).

Focus Group	Number of Vets	Male	Female	Predefined Criteria of Selection	Working Place (Federal State)
1	7	3	4	▪ Specialists at university hospital ▪ Capital city (Vienna) ▪ Well-equipped clinic ▪ High number of colleagues	Vienna
2	5	3	2	▪ Manager director of corporate clinic/private clinic owners ▪ Urban region/provincial cities ▪ Well-equipped referral clinic ▪ 5–18 employed veterinarians	Vienna, Lower Austria, Upper Austria, Vorarlberg, Styria region
3	4	1	3	▪ Specialists at referral clinics ▪ Provincial cities ▪ Well-equipped clinic ▪ Number colleagues > 3	Vienna, Lower Austria, Salzburg land, Vorarlberg
4	6	4	2	▪ General practitioners ▪ Capital city (Vienna) ▪ Small practices with basic equipment ▪ Self-employed (with 1–2 colleagues)	Vienna
5	5	1	4	▪ General practitioners ▪ Provincial cities/rural region ▪ Small practices with basic equipment ▪ Self-employed (with 1–2 colleagues)	Lower Austria, Upper Austria, Burgenland, Styria region
6	5	3	2^1^	▪ General practitioners ▪ Provincial cities/rural region ▪ Small practices with basic equipment ▪ Self-employed (with one colleague)	Carinthia, Salzburg land, Tyrol, Vorarlberg region

^1^ One of the female participants in Group 6 worked as a specialist at a referral clinic. She was wrongly invited to the focus group discussion among general practitioners due to the poorly maintained website which was accessed via the classified directory. This misclassification has been taken into consideration in the analysis and presentation of results in the present paper.

## Supplementary Materials

The following are available online at https://www.mdpi.com/2076-2615/9/5/241/s1, interview guide.
