# Supplementary material for: “Patients’ interests first, but … ”–Austrian Veterinarians’ Attitudes to Moral Challenges in Modern Small Animal Practice
Source: Animals (Basel). 2019 May 15;9(5):241. doi: 10.3390/ani9050241 (PMC6562789; doi:10.3390/ani9050241)
Supplement: Supplementary file 1 [file animals-09-00241-s001.pdf]

INTERVIEW GUIDE FOR FOCUS GROUPS 1 AND 3 on
"The Internal Morality of the Veterinary Profession in Modern Small Animal Medicine"

The present interview guide is valid for focus groups 1 and 3, which will take place as part of the project "The Internal Morality of the Veterinary Profession in modern small animal medicine". Focus groups 1 and 3 aim to reveal insights into the attitudes and views of practicing veterinarians, who are working in different fields of specialisation (oncology, surgery etc.) and are employed at the Vetmeduni, Vienna (focus group 1) or at an Austrian referral clinic (focus group 3).

Timeline:

- **Introduction** (5 min)
- **Presentation round:** Participants should introduce themselves and briefly report on their professional background (20 min)
- **Part 1:** General questions regarding patient care of small animal practitioners and owners expectations due to advanced diagnostics and therapy in small veterinary medicine (25 min)
- **Part 2:** Guiding the discussion towards advanced diagnostics and therapy in veterinary medicine and related uncertainties (30 min)

PAUSE (15 min)

- **Part 3:** Confronting participants with "provocative" headings and asking for comments and statements (30 min)
- **Part 4:** Presentation of case vignettes in order to explicate responses to different moral dilemmas occurring in the context of advanced veterinary diagnostics and therapy. Case vignettes will reveal the following aspects:
  1.) emotional bond/ overtreatment
  2.) disagreement with a colleague, who wants to push questionable treatment together with the animal owner
  3.) colleague, who wants to push high-risk treatment in order to try a new and highly complicated surgery method (35 min)

**Introduction:**<br>We would like to welcome you at [the Messerli Research Institute on the campus of the University of Veterinary Medicine, Vienna]. Thank you for participating in this group discussion, which will take place within the framework of a research project carried out by the Unit of Ethics and Human-Animal Studies and we are looking forward to the next approximately 2,5 hours.<br>First of all, I would like to introduce myself:<br>*[Herwig introduces himself]*<br>Furthermore, Svenja Springer is also present. She will make additional notes during the discussion, in order	**Introduction**

to record all relevant information and aspects arising in this discussion.<br>We will debate with further five groups in Austria. Due to the fact, that we cannot remember everything that is said and discussed, we will record the discourse on tape. All records are intended for our use, will not be passed on third parties and will be treated confidentially.<br>Before we will start with a presentation round, we would like to mention, that we are very interested in your thoughts, opinions and attitudes. We will start the discussion with very broad questions and will successively work together on the specific topic. Anything that seems important for you and comes to your mind is important for us. So we invite you to use this open space for discussion and there are no "wrong" answers.<br>But let´s start with a little presentation round where each of you have the possibility to introduce yourself with name and a briefly report on your professional background (Where are you working? How many colleagues working with you? Any specialisation?)<br><br>(20 min)	**Presentation round – every participant will introduce her-/himself by name/professional background/working environment/numbers of colleagues/field of specialisation**
**Part 1:**<br>**General questions regarding patient care of small animal practitioners and owner's expectations due to advanced diagnostics and therapy in small veterinary medicine.**<br><br>The daily tasks and work processes of practicing veterinarians are very complex and manifold. In order to get an overall impression of your day-to-day business we would like to ask you to take a short time and write down what are the three most important aspects for you as treating veterinarian regarding the care of your patients?<br>[3 minutes]<br><br>*The moderator asks each participant to tell what he/she has written down. We expect a variation of written aspects like act in the interest of the animal, patients' well-being, taking into account the care of the owner, best possible diagnosis and therapy for the patient, consultation and exchange with other colleagues etc. In order to create a discussion participants are not asked one after other, but rather the moderator asks in the round whether someone has listed other/different points that are important for him/her.*	**This part of the interview guide aims to provide an overall impression of important aspects regarding patient care in small animal practice (theme 1).**

Has anyone here written down other aspects or would like to comment on the aspects of the colleague?<br><br>*By means of this general question and the written aspects participants have the possibility to report on their daily working life in regard to patient care and related factors. The free conversation phase will provide a forum for diverse opinion and participants will have the opportunity to exchange their thoughts and ideas.*<br><br>*In addition, the moderator will ask to what extent the participants would weight the aspects according to their importance.*<br><br>To what extent would you weight the written aspects according to their importance? And why?<br><br>*We expect that through the weighting and discussion about the written aspects participants will reveal challenging situations, where they are confronted with conflicts of interests (e.g. acting in the best interest of the animal, but have to take into account the owners interests or the owner sets limitation due to financial reasons etc.). Against this background, the moderator asks questions to the plenary, when topics and issues related to challenging clients and clients' expectations occur:*<br><br>▪ You´ve told about challenging situation with clients. What kind of clients are challenging? And why?<br><br>*If participants do not mention challenging situations with clients the moderator will ask the following question as transition:*<br>▪ You've told about different aspects regarding patient care and their importance. To what extent do owners influence these aspects and lead to challenging situations?<br>▪ What kind of clients are challenging? And why?<br>▪ Are you confronted with increasing expectations from the owner's side due to the development of diagnostic and therapeutic possibilities in veterinary medicine? How do you deal with it?<br><br>(25 min)	**Participants have to prioritise interests of stakeholders within the described *trialogue* between veterinarian, animal and client.**<br><br>**Identification of**<br>**a) challenges related to clients and**<br>**b) uncertainties related to owners' expectations due to the development of diagnostic and therapeutic possibilities in veterinary medicine.**

<table>
<tr>
<td>

Part 2:
Guiding the discussion towards advanced diagnostics and therapy in veterinary medicine and possible emerging uncertainties.

In general, we expect that during the discussion about the aforementioned general questions the topic of advanced veterinary medicine was already mentioned or on the sidelines of discussion. Against this background, the opportunity exists to take up already mentioned facts, which lead to uncertainties in veterinarians' daily-working life. The aim of this theme is to identify possible uncertainties, which arise due to the modern veterinary medicine.

- What kind of technical device you would like to have in the clinic, where you are working that is currently not available? And why?

We talked about changing expectations from the owners' sides due to the development of veterinary medicine and advanced diagnostics and therapy.

- At which point or in which situation do you feel uncertain whether or nor not to exhaust all available diagnostic or therapeutic options for your patients' care? And why?
- Can you mention cases where you think that veterinarians go too far in diagnostics and therapy? And, can you name reasons for this?

Due to the fact, that advanced veterinary methods are not only linked to technical devices, the moderator will ask specifically if participants take up the following aspects during the discussion of both questions:

- *components related to the owner:*
    - *financial background*
    - *willingness*
    - *relational based factors (emotional bond etc.)*
    - *compliance*
- *components related to the patient:*
    - *technical feasibility exists, but the question of reasonableness arise*
    - *to what extent play criteria based reasons of the animal (e.g. species and age) a role*
- *components related to colleagues:*
    - *different knowledge base and expertise due to veterinarians specialisation lead to disagreements and different opinions*

</td>
<td>

This second part of the interview guide aims to direct the discussion towards the topic of advanced diagnostics and therapy in veterinary medicine (theme 2).

Identification of uncertainties related to the use of advanced diagnostics and therapy.

Identification of situation, where veterinarians retrospectively would state, that they have gone too far. Further, contextual factors can be identified, which may have influenced the decisions.

</td>
</tr>
</table>

regarding patient care<br><br>(30 min)<br>(total time frame: 75 min)	
**PAUSE** (15 min)	
**Part 3:**<br>**Confronting participants with provocative headings from popular scientific newspapers.**<br><br>After this short break we would like to present you the following headings, which were recently published in popular scientific newspapers.<br><br>*Participants will be shown three different headliners, which reveal the issue of advanced diagnostics and therapy in veterinary medicine in a provocative way:*<br><br>1. Luxury Medicine for Pet Animals: Dogs, do you want to live forever? (Reference: SPIEGEL Online)<br>2. Sick pet animals between high-tech and liver sausage… (Reference: WELT N24)<br>3. Limitless veterinary medicine? More and more quality of life is crucial. (Reference: Mein Haustier - Gesundheitsmagazin für Tiere)<br><br>*By means of these provocative headings, we expect that participants have to take up a position, which is based on their normative conviction on the one hand. This approach enables an explication of moral dimensions. On the other hand, we expect that contextual factors like different working environment or field of specialisation lead to different argumentations.*<br>*Following questions can be used to locate possible problem areas and fields of tension:*<br><br>The continuous and rapid development in veterinary medicine is presented here in a very provocative way.<br>We would like to ask you to comment on these statements.<br>▪ In general, are there any diagnostic or therapeutic procedures in small animal medicine, which are going too far? And Why?<br>▪ [Are you confronted with different attitudes on the topic within you working environment? → Focus group 3]<br><br>(30 min)	**Participants are asked to comment on presented provocative headings (theme 3).**<br><br>**By means of headings, the discussion is raised from the veterinarian-owner-animal context to a social context or discourse. Different attitudes and associated uncertainties of veterinarians will be vocalized and enable an explication of**<br>**1) attitudes towards this headings as well as**<br>**2) possible leading norms and principles arising in their argumentation (e.g. avoiding suffering, quality of life).**<br><br>**Further, discussion about headings and asked questions can explicate possible different attitudes towards advanced medthods in veterinary medicine in relation to specific fields of specialisation (e.g. oncology, anaesthesia).**

**Part 4:**<br>**Presentation of case vignettes in order to explicate responses to different moral dilemmas occurring in the context of advanced veterinary diagnostics and therapy.**<br>*(see file: Case_Vignettes_Theme_4_FG_eng)*<br><br>*In order to reveal how veterinarians are torn between the medical feasibility, ethical requirements and contextual factors, participants will be confronted with several case vignettes focusing on specific aspects:*<br>*1.) emotional bond/ overtreatment*<br>*2.) disagreement with a colleague, who wants to push questionable treatment together with the animal owner*<br>*3.) colleague, who wants to push high-risk treatment in order to try a new and highly complicated surgery method*<br><br>*The case vignettes constitute the basic content and offer incentive for the discussion including relevant contextual factors. Uncertainties, differing opinions and attributed responsibilities can be make visible within the different given frameworks/situations.*<br><br>As a last point, we would like to discuss with you the following case vignettes and kindly ask you to comment on them.<br><br>*Relevant topics of each case vignettes should be discussed. The moderator should pay attention, that the specific aspects 1.) – 3.) are subjects of discussion. This approach enables a systematic investigation of how veterinarians assess and handle such situations in consideration of relevant contextual factors.*<br><br>(35 min)<br>(total time frame including break: 155 min)	**Designed case vignettes address factors, which are of relevance in the context of advanced veterinary medicine. It can be identify 1) how veterinarians handle such situations and 2) to what extent do factors relating to the animal, client's characteristics, the veterinarian as well as working environment determine decision-making processes.**<br><br>**This approach allows drawing a conclusion on the IMVM, which will be constructed in response to moral dilemmas.**
**End:**<br>Finally, we would like to thank you for your time, effort and cooperation. This was a very fruitful and valuable discussion. Now, we would like to invite you to a get-together in order to end the focus group in a relaxed atmosphere.<br><br>Thank you very much!	**Acknowledgment to all participants for their time, effort and cooperation. Followed by a closing with drinks and snacks (get-together).**

INTERVIEW GUIDE FOR FOCUS GROUP 2 on
"The Internal Morality of the Veterinary Profession in Modern Small Animal Medicine"

The present interview guide is valid for focus group 2, which will take place as part of the project "The Internal Morality of the Veterinary Profession in the age of modern small animal medicine". Focus group 2 aims to reveal insights into the attitudes and views of managers and clinic owners of Austrian referral clinics within the specific context of advanced diagnostics and therapy in small animal practice. Compared to focus group 3, the discussion in focus group 2 will be more focus on the role as service provider.

Timeline:

- **Introduction** (5 min)
- **Presentation round:** Participants should introduce themselves and briefly report on their professional background (20 min)
- **Part 1:** General questions regarding patient care of clinic owner/manager as well as small animal practitioners and owners expectations due to advanced diagnostics and therapy in small veterinary medicine (25 min)
- **Part 2:** Guiding the discussion towards advanced diagnostics and therapy in veterinary medicine and related uncertainties (30 min)

PAUSE (15 min)

- **Part 3:** Confronting participants with "provocative" headings and asking for comments and statements (30 min)
- **Part 4:** Presentation of case vignettes in order to explicate responses to different moral dilemmas occurring in the context of advanced veterinary diagnostics and therapy. Case vignettes will reveal the following aspects:
  1.) emotional bond/ overtreatment
  2.) disagreement with a colleague, who wants to push questionable treatment together with the animal owner
  3.) colleague, who wants to push high-risk treatment in order to try a new and highly complicated surgery method (35 min)

**Introduction:**<br>We would like to welcome you at [the Messerli Research Institute on the campus of the University of Veterinary Medicine, Vienna]. Thank you for participating in this group discussion, which will take place within the framework of a research project carried out by the Unit of Ethics and Human-Animal Studies and we are looking forward to the next approximately 2,5 hours.<br>First of all, I would like to introduce myself:<br>*[Herwig introduces himself]*<br>Furthermore, Svenja Springer is also present. She will make additional notes during the discussion, in order	**Introduction**

to record all relevant information and aspects arising in this discussion.<br>We will debate with further five groups in Austria. Due to the fact, that we cannot remember everything that is said and discussed, we will record the discourse on tape. All records are intended for our use, will not be passed on third parties and will be treated confidentially.<br>Before we will start with a presentation round, we would like to mention, that we are very interested in your thoughts, opinions and attitudes. We will start the discussion with very broad questions and will successively work together on the specific topic. Anything that seems important for you and comes to your mind is important for us. So we invite you to use this open space for discussion and there are no “wrong” answers.<br>But let´s start with a little presentation round where each of you have the possibility to introduce yourself with name and a briefly report on your professional background (Where are you working? How many veterinarians are employed in the clinic? Are you still working as practicing veterinarian?)<br><br>(20 min)	**Presentation round – every participant will introduce her-/himself by name/professional background/working environment/number of staff members/field of specialisation**
**Part 1:**<br>**General questions regarding patient care of small animal practitioners [respectively clinic owner/manager] and owner’s expectations due to advanced diagnostics and therapy in small veterinary medicine.**<br><br>The daily tasks and work processes of practicing veterinarians are very complex and manifold. Beside your work as veterinarian you are managing the clinical operations. In order to get an overall impression of your day-to-day business we would like to ask you to take a short time and write down what are the three most important aspects for you regarding the care of patients?<br>[3 minutes]<br><br>*The moderator asks each participant to tell what he/she has written down. We expect a variation of written aspects like act in the interest of the animal, patients’ well-being, taking into account the care of the owner, best possible diagnosis and therapy for the patient, consultation and exchange with other colleagues/staff members etc. In order to create a*	**This part of the interview guide aims to provide an overall impression of important aspects regarding patient care in small animal practice (theme 1).**

discussion participants are not asked one after other, but rather the moderator asks in the round whether someone has listed other/different points that are important for him/her.<br><br>Has anyone here written down other aspects or would like to comment on the aspects of the colleague?<br><br>*By means of this general question and the written aspects participants have the possibility to report on their daily working life in regard to patient care and related factors. The free conversation phase will provide a forum for diverse opinion and participants will have the opportunity to exchange their thoughts and ideas.*<br><br>*In addition, the moderator will ask to what extent the participants would weight the aspects according to their importance.*	
To what extent would you weight the written aspects according to their importance? And why?<br><br>*We expect that through the weighting and discussion about the written aspects participants will reveal challenging situations, where they are confronted with conflicts of interests (e.g. acting in the best interest of the animal, but have to take into account the owners interests or the owner sets limitation due to financial reasons etc.). Against this background, the moderator asks questions to the plenary, when topics and issues related to challenging clients and clients' expectations occur:*<br><br>▪ You´ve told about challenging situation with clients. What kind of clients are challenging? And why?<br><br>*If participants do not mention challenging situations with clients the moderator will ask the following question as transition:*<br>▪ You've told about different aspects regarding patient care and their importance. To what extent do owners influence these aspects and lead to challenging situations?<br>▪ What kind of clients are challenging? And why?<br>▪ Are you confronted with increasing expectations from the owner's side due to the development of diagnostic and therapeutic possibilities in veterinary medicine? How do you deal with it as clinic owner/manager?<br>▪ Do you prioritise owners' expectations differently in your position as clinic	**Participants have to prioritise interests of stakeholders within the described *trialogue* between veterinarian, animal and client.**<br><br>**Identification of**<br>**a) challenges related to clients and**<br>**b) uncertainties related to owners' expectations due to the development of diagnostic and therapeutic possibilities in veterinary medicine.**<br>**Further, it should be identified if the position as clinic owner/manger leads to a different attitude towards owners.**

owner/manger?<br><br>(25 min)	
**Part 2:**<br>**Guiding the discussion towards advanced diagnostics and therapy in veterinary medicine and possible emerging uncertainties.**<br><br>*In general, we expect that during the discussion about the aforementioned general questions the topic of advanced veterinary medicine was already mentioned or on the sidelines of discussion. Last questions of part 1 already addressed a possible change of owners' expectations regarding the treatment of their animal. The following question shall identify different reasons why clinic owners buy new technical devices for their clinic. Answers can reveal arguments like offering best diagnostic and treatment options for the patient, providing animal owner more veterinary treatment options, make more money by providing advanced service or because of growing competition with other clinics etc.*<br><br>We talked about changing expectations from the owners' sides due to the development of veterinary medicine and advanced diagnostics and therapy.<br><br>▪ When did you buy the last technical device for your clinic? And why?<br><br>*Further, the discussion shall be directed towards emerging uncertainties, which arise in the context of advanced veterinary medicine. Against this background, the opportunity exists to take up already mentioned facts, which lead to uncertainties in clinic owners' daily-working life. The aim of this theme is to identify possible uncertainties, which arise due to the modern.*<br><br>▪ At which point/in which situation do you feel uncertain whether or nor not to exhaust all available diagnostic or therapeutic options for patients' care at your clinic? And why?<br><br>▪ Can you name cases where you think veterinarians go too far in diagnostics or therapy? And, can you name reasons for this?<br><br>*Due to the fact, that advanced veterinary methods is not only linked to technical devices, the moderator will ask specifically if participants take up the following aspects during the discussion of the last questions:*	**This second part of the interview guide aims to direct the discussion towards the topic of advanced diagnostics and therapy in veterinary medicine (theme 2).**<br><br>**Identification of reasons for buying and providing new technical devices in referral clinics.**<br><br>**Identification of uncertainties related to the use of advanced diagnostics and therapy.**<br><br>**Identification of situation, where veterinarians retrospectively would state, that they have gone too far. Further, contextual factors can be identified, which may have influenced**

<table>
<tr>
<td>

- *components related to the owner:*
  - *financial background*
  - *willingness*
  - *relational based factors (emotional bond etc.)*
  - *compliance*
- *components related to the patient:*
  - *technical feasibility exists, but the question of reasonableness arise*
  - *to what extent play criteria based reasons of the animal (e.g. species and age) a role*
- *components related to colleagues/staff-members:*
  - *different knowledge base and expertise due to veterinarians specialisation lead to disagreements and different opinions regarding patient care*

(30 min)
(total time frame: 75 min)

</td>
<td>**the decisions.**</td>
</tr>
<tr>
<td>**PAUSE** (15 min)</td>
<td></td>
</tr>
<tr>
<td>

Part 3:
Confronting participants with provocative headings from popular scientific newspapers.

After this short break we would like to present you the following headings, which were recently published in popular scientific newspapers.

Participants will be shown three different headliners, which reveal the use of high-tech in veterinary medicine in a provocative way:

1. Luxury Medicine for Pet Animals: Dogs, do you want to live forever? (Reference: SPIEGEL Online)
2. Sick pet animals between high-tech and liver sausage… (Reference: WELT N24)
3. Limitless veterinary medicine? More and more quality of life is crucial. (Reference: Mein Haustier - Gesundheitsmagazin für Tiere)

By means of these provocative headings, we expect that participants have to take up a position, which is based on their normative conviction on the one hand. This approach enables an explication of moral dimensions. On the other hand, we expect that contextual factors like different working environment or field of specialisation lead to different argumentations.
Following questions can be used to locate possible problem areas and fields of tension:

The continuous and rapid development in veterinary

</td>
<td>

Participants are asked to comment on the provocative headings (theme 3).

By means of headings, the discussion is raised from the veterinarian-owner-animal context to a social context or discourse. Different attitudes and associated uncertainties of veterinarians will be vocalized and enable an explication of
1) attitudes towards this headings as well as
2) possible leading norms and principles arising in their argumentation (e.g. avoiding suffering, quality of life).

</td>
</tr>
</table>

medicine is presented here in a very provocative way. We would like to ask you to comment on these statements.<br>▪ As clinic owner, do you feel under pressure to be constantly up to date with the latest technology?<br>▪ In general, are there any diagnostic or therapeutic procedures, which are going too far? And Why?<br><br>(30 min)	**Further, discussion about headings and questions can explicate how clinic owners assess advanced veterinary methods in regard to service provision.**
**Part 4:**<br>**Presentation of case vignettes in order to explicate responses to different moral dilemmas occurring in the context of advanced veterinary diagnostics and therapy.**<br>*(see file: Case_Vignettes_Theme_4_FG_eng)*<br><br>*In order to reveal how clinic owner are torn between the medical feasibility, ethical requirements and contextual factors, participants will be confronted with several case vignettes focusing on specific aspects:*<br>*1.) emotional bond/ overtreatment*<br>*2.) disagreement with a colleague, who wants to push questionable treatment together with the animal owner*<br>*3.) colleague, who wants to push high-risk treatment in order to try a new and highly complicated surgery method*<br><br>*The case vignettes constitute the basic content and offer incentive for the discussion including relevant contextual factors. Uncertainties, differing opinions and attributed responsibilities can be make visible within the different given frameworks/situations.*<br><br>As a last point, we would like to discuss with you the following case vignettes and kindly ask you to comment on them.<br><br>*Relevant topics of each case vignettes should be discussed. The moderator should pay attention, that the specific aspects 1.) – 3.) are subjects of discussion. This approach enables a systematic investigation of how veterinarians assess and handle such situations in consideration of relevant contextual factors.*<br><br>(35 min)<br>(total time frame including break: 155 min)	**Designed case vignettes address factors, which are of relevance in the context of advanced veterinary medicine. It can be identify 1) how they handle such situations and 2) to what extent do factors relating to the animal, client's characteristics, the clinic owner as well as working environment determine decision-making processes.**<br><br>**This approach allows drawing a conclusion on the IMVM, which will be constructed in response to moral dilemmas.**

**End:**<br>Finally, we would like to thank you for your time, effort and cooperation. This was a very fruitful and valuable discussion. Now, we would like to invite you to a get-together in order to end the focus group in a relaxed atmosphere.<br><br>Thank you very much!	**Acknowledgment to all participants for their time, effort and cooperation. Followed by a closing with drinks and snacks (get-together).**

INTERVIEW GUIDE FOR FOCUS GROUPS 4, 5 AND 6 on
"The Internal Morality of the Veterinary Profession in Modern Small Animal Medicine"

The present interview guide is valid for focus groups 4 to 6, which will take place as part of the project "The Internal Morality of the Veterinary Profession in the age of modern small animal medicine". Focus groups 4 to 6 aim to reveal insights into the attitudes and views of self-employed practicing veterinarians in Austria within the specific context of advanced diagnostics and therapy in small animal practice.

Timeline:

- **Introduction** (5 min)
- **Presentation round:** Participants should introduce themselves and briefly report on their professional background (20 min)
- **Part 1:** General questions regarding patient care of small animal practitioners and owners expectations due to advanced diagnostics and therapy in small veterinary medicine (25 min)
- **Part 2:** Guiding the discussion towards advanced diagnostics and therapy in veterinary medicine and related uncertainties (30 min)

PAUSE (15 min)

- **Part 3:** Confronting participants with "provocative" headings and asking for comments and statements (30 min)
- **Part 4:** Presentation of case vignettes in order to explicate responses to different moral dilemmas occurring in the context of advanced veterinary diagnostics and therapy. Case vignettes will reveal the following aspects:
  1.) emotional bond/ overtreatment
  2.) disagreement with a colleague, who wants to push questionable treatment together with the animal owner
  3.) colleague, who wants to push high-risk treatment in order to try a new and highly complicated surgery method (35 min)

**Introduction:**<br>We would like to welcome you at [the Messerli Research Institute on the campus of the University of Veterinary Medicine, Vienna]. Thank you for participating in this group discussion, which will take place within the framework of a research project carried out by the Unit of Ethics and Human-Animal Studies and we are looking forward to the next approximately 2,5 hours.<br>First of all, I would like to introduce myself:<br>*[Herwig introduces himself]*<br>Furthermore, Svenja Springer is also present. She will make additional notes during the discussion, in order to record all relevant information and aspects arising in	**Introduction**

this discussion.<br>We will debate this topic with further five groups in Austria. Due to the fact, that we cannot remember everything that is said and discussed, we will record the discourse on tape. All records are intended for our use, will not be passed on third parties and will be treated confidentially.<br>Before we will start with a presentation round, we would like to mention, that we are very interested in your thoughts, opinions and attitudes. We will start the discussion with very broad questions and will successively work together on the specific topic. Anything that seems important for you and comes to your mind is important for us. So we invite you to use this open space for discussion and there are no “wrong” answers.<br>But let´s start with a little presentation round where each of you have the possibility to introduce yourself with name and a briefly report on your professional background and where are you working.<br>(Where are you working? How many colleagues working with you? How long have you been working as veterinarian? How long have you been owning your practice?)<br><br>(20 min)	**Presentation round – every participant will introduce her-/himself by name/professional background/working environment**
**Part 1:**<br>**General questions regarding patient care of small animal practitioners and owner’s expectations due to advanced diagnostics and therapy in small veterinary medicine.**<br><br>The daily tasks and work processes of practicing veterinarians are very complex and manifold. In order to get an overall impression of your day-to-day business we would like to ask you to take a short time and write down what are the three most important aspects for you regarding the care of your patients?<br>[3 minutes]<br><br>*The moderator asks each participant to tell what he/she has written down. We expect a variation of written aspects like act in the interest of the animal, patients’ well-being, taking into account the care of the owner, best possible diagnosis and therapy for the patient, consultation and exchange with other colleagues etc. In order to create a discussion participants are not asked one after other, but rather the moderator asks in the round whether someone has listed*	**This part of the interview guide aims to provide an overall impression of important aspects regarding patient care in small animal practice (theme 1).**

other/different points that are important for him/her.<br><br>Has anyone here written down other aspects or would like to comment on the aspects of the colleague?<br><br>*By means of this general question and the written aspects participants have the possibility to report on their daily working life in regard to patient care and related factors. The free conservation phase will provide a forum for diverse opinion and participants will have the opportunity to exchange their thoughts and ideas.*<br><br>*In addition, the moderator will ask to what extent the participants would weight the aspects according to their importance.*<br><br>To what extent would you weight the written aspects according to their importance? And why?<br><br>*We expect that through the weighting and discussion about the written aspects participants will reveal challenging situations, where they are confronted with conflicts of interests (e.g. acting in the best interest of the animal, but have to take into account the owners interests or the owner sets limitation due to financial reasons etc.). Against this background, the moderator asks questions to the plenary, when topics and issues related to challenging clients and clients' expectations occur:*<br><br>▪ You´ve told about challenging situation with clients. What kind of clients are challenging? And why?<br><br>*If participants do not mention challenging situations with clients the moderator will ask the following question as transition:*<br>▪ You've told about different aspects regarding patient care and their importance. To what extent do owners influence these aspects and lead to challenging situations?<br>▪ What kind of clients are challenging? And why?<br>▪ Are you confronted with increasing expectations from the owner's side due to the development of diagnostic and therapeutic possibilities in veterinary medicine? How do you deal with it?<br><br>(25 min)	**Participants have to prioritise interests of stakeholders within the described *trialogue* between veterinarian, animal and client.**<br><br>**Identification of**<br>**a) challenges related to clients and**<br>**b) uncertainties related to owners' expectations due to the development of diagnostic and therapeutic possibilities in veterinary medicine.**

**Part 2:**<br>**Guiding the discussion towards advanced diagnostics and therapy in veterinary medicine and possible emerging uncertainties.**	
In general, we expect that during the discussion about the aforementioned general questions the topic of advanced veterinary medicine was already mentioned or on the sidelines of discussion. The following question shall identify different reasons why self-employed veterinarians buy new technical devices for their practice. Answers can reveal arguments like offering best diagnostic and treatment options for the patient, providing animal owner more veterinary treatment options, make more money by providing advanced service or because of growing competition with other practices or clinics etc.<br><br>We talked about changing expectations from the owners' sides due to the development of veterinary medicine and advanced diagnostics and therapy.	**This second part of the interview guide aims to direct the discussion towards the topic of advanced diagnostics and therapy in veterinary medicine (theme 2).**
▪ What kind of technical device you would like to buy for your practice that you don't have currently? And why?<br><br>*Further, the discussion shall be directed towards emerging uncertainties, which arise in the context of advanced veterinary medicine. Against this background, the opportunity exists to take up already mentioned facts, which lead to uncertainties in clinic owners' daily-working life. The aim of this theme is to identify possible uncertainties, which arise due to the modern.*	**Identification of reasons for buying and providing new technical devices in practice.**
▪ At which point/in which situation do you feel uncertain whether or nor not to exhaust all available diagnostic or therapeutic options for patients' care at your clinic? And why?	**Identification of uncertainties related to the use of advanced diagnostics and therapy.**
▪ Can you name cases where you think veterinarians go too far in diagnostics and therapy? And, can you name reasons for this?<br><br>*Due to the fact, that advanced veterinary methods is not only linked to technical devices, the moderator will ask specifically if participants take up the following aspects during the discussion of the last questions:*<br><br>▪ *components related to the owner:*<br>o *financial background*<br>o *willingness*<br>o *relational based factors (emotional bond*	**Identification of situation, where veterinarians retrospectively would state, that they have gone too far. Further, contextual factors can be identified, which may have influenced the decisions.**

etc.)<br>o *compliance*<br>▪ *components related to the patient:*<br>o *technical feasibility exists, but the question of reasonableness arise*<br>o *to what extent play criteria based reasons of the animal (e.g. species and age) a role*<br>▪ *components related to colleagues/staff-members:*<br>o *different knowledge base and expertise due to veterinarians specialisation lead to disagreements and different opinions regarding patient care*<br><br>(30 min)<br>(total time frame: 75 min)	
**PAUSE** (15 min)	
**Part 3:**<br>**Confronting participants with provocative headings from popular scientific newspapers.**<br><br>After this short break we would like to present you the following headings, which were recently published in popular scientific newspapers.<br><br>*Participants will be shown three different headliners, which reveal the issue of advanced diagnostics and therapy in veterinary medicine in a provocative way:*<br>1. Luxury Medicine for Pet Animals: Dogs, do you want to live forever? (Reference: SPIEGEL Online)<br>2. Sick pet animals between high-tech and liver sausage… (Reference: WELT N24)<br>3. Limitless veterinary medicine? More and more quality of life is crucial. (Reference: Mein Haustier - Gesundheitsmagazin für Tiere)<br><br>*By means of these provocative headings, we expect that participants have to take up a position, which is based on their normative conviction on the one hand. This approach enables an explication of moral dimensions. On the other hand, we expect that contextual factors like different working environment or field of specialisation lead to different argumentations.*<br>*Following questions can be used to locate possible problem areas and fields of tension:*<br><br>The continuous and rapid development in veterinary medicine is presented here in a very provocative way.<br>We would like to ask you to comment on these statements.<br>▪ As self-employed veterinarian, do you fell	**Participants are asked to comment on the provocative headings (theme 3).**<br><br>**By means of headings, the discussion is raised from the veterinarian-owner-animal context to a social context or discourse. Different attitudes and associated uncertainties of veterinarians will be vocalized and enable an explication of**<br>**1) attitudes towards this headings as well as**<br>**2) possible leading norms and principles arising in their argumentation (e.g. avoiding suffering, quality of life).**<br><br>**Further, discussion about**

under pressure to be constantly up to date with the latest diagnostics and treatment options?<br>▪ In general, are there any diagnostic or therapeutic procedures in small animal medicine, which are going too far? And Why?<br>▪ How do you perceive and deal with different attitudes among the veterinary profession?<br><br>*We expect that participants of focus groups 4 to 6 are doing standard procedures and they refer patients to clinics in case of patients with difficult diagnosis/therapy plans or with the need of several technique devices.*<br><br>▪ During treatment process of patients, at what point you refer a patient to a referral clinic? And why?<br>▪ Has it ever happened to you that you lost client, because you refer the patient to a clinic? If so, how do you deal with such situations?<br><br>*By means of this question, we can explicate the following aspect in more depth: Whether veterinarians are not able to do such interventions, because of the available technique in their practice, or they do not want to do certain kind of procedures, because of their general attitude towards advanced veterinary diagnostics and therapy. A further aspect can be that veterinarians are unwilling to refer patients to clinics, because they are afraid that they will loose the owner.*<br>(30 min)	**headings and questions can explicate how self-employed veterinarians assess advanced veterinary methods in regard to service provision.**
**Part 4:**<br>**Presentation of case vignettes in order to explicate responses to different moral dilemmas occurring in the context of advanced veterinary diagnostics and therapy.**<br>*(see file: Case_Vignettes_Theme_4_FG_eng)*<br><br>*In order to reveal how self-employed veterinarians are torn between the medical feasibility, ethical requirements and contextual factors, participants will be confronted with several case vignettes focusing on specific aspects:*<br>*1.) emotional bond/ overtreatment*<br>*2.) disagreement with a colleague, who wants to push questionable treatment together with the animal owner*<br>*3.) colleague, who wants to push high-risk treatment in order to try a new and highly complicated surgery method*<br><br>*The case vignettes constitute the basic content and offer incentive for the discussion including relevant contextual*	**Designed case vignettes address factors, which are of relevance in the context of advanced veterinary medicine. It can be identify 1) how they handle such situations and 2) to what extent do factors relating to the animal, client's characteristics, the self-employed veterinarian as well as working environment determine decision-making processes.**<br><br>**This approach allows drawing a conclusion on**

factors. Uncertainties, differing opinions and attributed responsibilities can be make visible within the different given frameworks/situations.<br><br>As a last point, we would like to discuss with you the following case vignettes and kindly ask you to comment on them.<br><br>*Relevant topics of each case vignettes should be discussed. The moderator should pay attention, that the specific aspects 1.) – 3.) are subjects of discussion. This approach enables a systematic investigation of how veterinarians assess and handle such situations in consideration of relevant contextual factors.*<br>(35 min)<br>(total time frame including break: 155 min)	**the IMVM, which will be constructed in response to moral dilemmas.**
**End:**<br>Finally, we would like to thank you for your time, effort and cooperation. This was a very fruitful and valuable discussion. Now, we would like to invite you to a get-together in order to end the focus group in a relaxed atmosphere.<br><br>Thank you very much!	**Acknowledgment to all participants for their time, effort and cooperation. Followed by the closing with drinks and snacks (get-together).**

LIST OF CODES

**CATEGORY 1:** CHALLENGING CLIENTS and EXPECTATIONS	**CC**	**Category 1 includes a) challenges related to clients and b) clients' expectations due to the development of diagnostic and therapeutic possibilities in small animal medicine.**
CC: characteristics - reasons	CC-CHARAC	Identified<br>a) characteristics or types of challenging clients including reported character attributes, behaviours and certain ways of acting or thinking;<br>b) reasons why these clients are described as challenging and leads to dissonance between veterinarian and client (e.g. difficult to manage in daily routine (time management) or psychologically challenging),
CC: dealing	CC-DEAL	Identified ways of dealing with challenging clients, such as addressing specific points directly, trying to ignore them or taking specific measures to get rid of the client.
CC: expectations and explanation	CC-EXPEC	Identified<br>a) expectations of clients due to the development of diagnostic and therapeutic possibilities in small animal practice including described expectations of clients towards the veterinary profession in general, towards veterinarian in individual cases or possible inappropriate attitudes of clients towards new possibilities in veterinary medicine;<br>b) explanation for increasing expectations (e.g. clients compare veterinary medicine with progresses and possibilities in human medicine, further sources of information (television, newspapers, internet, social media etc.) or status of the animal (family member)).
**CATEGORY 2:** ADAVANCED VETERINARY MEDICINE	**AVM**	**Category 2 refers to general aspects related to advanced diagnostic and therapeutic possibilities in small animal practice.**
AVM: medical technical devices	AVM-TEC	Technical devices veterinarians would like to use for patient care, e.g. MRI, CT or digital X-ray machine.
AVM: medical technical devices and reasons	AVM-TEC/REAS	Reasons why veterinarians would choose the technical device including aspects like facilitation in course of diagnosis or therapy for veterinarian (better quality, faster, easier to handle), improvement of patient care or client demand.
AVM: other aspects and specialisation	AVM-ASP	Other aspects of advanced veterinary medicine, which give an indication of what veterinarians mean when they talk about advanced veterinary medicine, aspects related to the veterinarians

		field of specialisation (e.g. ordinary treatment, high sophisticated treatment (special surgeries, which is not only related to technical devices and advanced specialised knowledge) as well as implementation of tools, checklists and lists for quality management due to the development of veterinary medicine.
AVM: changes, implementation and effects	AVM-CHAN	Identified<br>a) occurring changes for practicing veterinarian due to advanced medical methods (e.g. MRI, digital X-Ray, CT);<br>b) implementation of advanced veterinary methods (not only technical devices but rather sophisticated techniques for surgery etc.)<br>c) indices of impact of advanced veterinary medicine on I) animal patient, II) veterinarian, III) client and IV) profession in general (positive as well as negative impacts)
AVM: challenges	AVM-CHAL	Identified emerging challenges for practicing veterinarians due to advanced veterinary medicine and indices of impact of challenges on veterinarians.
AVM: dealing	AVM-DEAL	How veterinarians deal with challenges (*conceptual* [veterinarians recognize fields of tension, but react rationally] versus *emotional* [veterinarians not only recognize the challenging situation but rather this field of tension stressed them out]
AVM: attitudes towards AVM	AVM-ATT	Identified ways of thinking and feeling about the development of advanced veterinary medicine.
**CATEGORY 3:**<br>**AN**IMAL-BASED FACTORS	**AN**	**Category 3 refers to aspects related to the animal patient.**
AN: capacities and effects	AN-CHARAC	Identified<br>a) characteristics, attributes and nature of the animal (species, age etc.),<br>b) indices of impact of animal's attributes and characteristics on decision-making processes.
AN: guiding principles	AN-PRIN	Statements related to animals' wellbeing, avoidance of suffering and protect the patients' life as guiding principles within decision-making processes.
**CATEGORY 4:**<br>**CL**IENT-BASED FACTORS	**CL**	**Category 4 refers to aspects related to the client.**
CL: financial factors and effects	CL-FIN	Identified<br>a) components related to financial factors (e.g. client not able to pay, client not willing pay, expensive treatment costs)

		b) indices of impact of financial factors on decision-making processes (e.g. diagnosis, therapy, pet-insurance).
CL: emotional factors and effects	CL-EMO	Identified<br>a) components related to emotional factors (strong/weak emotional attachment, status of the animal) and<br>b) indices of impact of emotional factors on decision-making processes (e.g. overtreatment).
CL: living condition and effects	CM-LIV	Identified<br>a) components related to client's living condition (e.g. time problems, changes in housing, troubled family situation, decease spouse, housing situations= living environment)<br>b) indices of impact of client's living condition on decision-making processes.
**CATEGORY 5:**<br>**VET**ERINARIAN-BASED FACTORS	**VET**	**Category 5 refers to aspects related to the veterinarian.**
VET: specialisation, competences, experiences and effects	VET-SPEC	Identified<br>a) aspects related to the field of specialisation of veterinarians, experiences respectively competences (medical/technical) and<br>b) indices of impact of specialisation and associated competences on decision-making processes.
VET: service provider and effects	VET-SERV	Identified<br>a) aspects related to the position as service provider and veterinary practice as "service sector" (e.g. acting in the interest of the client, clients satisfaction) and<br>b) indices of impact of service provision on decision-making processes.
VET: position/functional area and effects	VET-POS	Identified<br>a) aspects related to different hierarchical position and functional areas in veterinary clinics (specialized veterinarians, clinic owner/manager) and small animal practices practicing veterinarians) and<br>b) indices of impact of position and different functional areas on decision-making processes.
VET: advocate position and effects	VET-ADVOC	Identified<br>a) aspects in which veterinarians reflect on their role and on the question of their position as advocate, focus on the interests of the animal<br>b) indices of impacts of advocate position on decision-making processes.

VET: relationship between veterinarian and client	VET-REL-CL	Identified<br>a) aspects related to the relationship between the veterinarian and his/her client (empathy, trust, respect, time, reliability, respectability etc.) and<br>b) indices of impacts of the relationship on decision-making processes
VET: economical aspects and effects	VET-ECON	Identified<br>a) aspects related to economical aspects of veterinary practice veterinarians/clinics need to make money to make a living and/or diagnostics and therapy just because of monetary aspects<br>b) indices of impact of economical aspects.
**CATEGORY 6:**<br>**COL**LEAGUES-BASED FACTORS	**COL**	**Category 6 refers to components and aspects related to colleagues and possible disagreements.**
COL: interaction	COL-INT	Identified<br>aspects related to interactions between veterinarians and their colleagues (in the same practice or clinic/ veterinarians working in small animal practices and veterinarians working in referral clinics/ veterinarians working both in small animal practices) in a technical sense (referral of patients, get in contact with colleagues)
COL: relationship	COL-REL	Identified<br>aspects related to relationships between veterinarians and their colleagues (in the same practice or clinic/ veterinarians working in small animal practices and veterinarians working in referral clinics/ veterinarians working both in small animal practices).
COL: disagreement and effects	COL-DIS	Identified<br>a) aspects related to disagreement among veterinarians (in the same practice or clinic/ veterinarians working in small animal practices and veterinarians working in referral clinics/ veterinarians working both in small animal practices)<br>b) indices of impacts of disagreements on decision-making processes.
**CATEGORY 7:**<br>**VET-STRATEGIES to deal with challenges**	**STRAT**	
STRAT: consultant	STRAT-COUN	Identified<br>a) aspects which mainly refer to the veterinarian's role as consultant during challenging

		decision-making processes;<br>b) indices of impacts of identified aspects on decision-making
STRAT: psychologist	STRAT-PSYCH	Identified<br>a) aspects which mainly refer to the veterinarian’s role as “psychologist” during challenging decision-making processes;<br>b) indices of impacts of identified aspects on decision-making
STRAT: advocate	STRAT-ADVOC	Identified<br>a) aspects which mainly refer to the veterinarian’s role as advocate during challenging decision-making processes;<br>b) indices of impacts of identified aspects on decision-making
STRAT: scientist	STRAT-SCIEN	Identified<br>a) aspects which mainly refer to the veterinarian’s role as scientist during challenging decision-making processes;<br>b) indices of impacts of identified aspects on decision-making
